# Cooperation between artificial intelligence and endoscopists for diagnosing invasion depth of early gastric cancer

**DOI:** 10.1007/s10120-022-01330-9

**Published:** 2022-08-30

**Authors:** Atsushi Goto, Naoto Kubota, Jun Nishikawa, Ryo Ogawa, Koichi Hamabe, Shinichi Hashimoto, Hiroyuki Ogihara, Yoshihiko Hamamoto, Hideo Yanai, Osamu Miura, Taro Takami

**Affiliations:** 1 Department of Gastroenterology and Hepatology, Yamaguchi University Graduate School of Medicine, Minami-kogushi 1-1-1, Ube, Yamaguchi 755-8505 Japan; 2 Department of Laboratory Science, Yamaguchi University Graduate School of Medicine, Ube, Yamaguchi Japan; 3 National Institute of Technology, Tokuyama College, Shunan, Yamaguchi Japan; 4 Division of Electrical, Electronic and Information Engineering, Yamaguchi University Graduate School of Sciences and Technology for Innovation, Ube, Yamaguchi Japan; 5 Department of Clinical Research, National Hospital Organization Kanmon Medical Center, Shimonoseki, Yamaguchi Japan; 6 Department of Surgery, Hofu Institute of Gastroenterology, Hofu, Yamaguchi Japan

**Keywords:** Early gastric cancer, Invasion depth, AI classifier

## Abstract

**Background and study aims:**

The diagnostic ability of endoscopists to determine invasion depth of early gastric cancer is not favorable. We designed an artificial intelligence (AI) classifier for differentiating intramucosal and submucosal gastric cancers and examined it to establish a diagnostic method based on cooperation between AI and endoscopists.

**Patients and methods:**

We prepared 500 training images using cases of mainly depressed-type early gastric cancer from 250 intramucosal cancers and 250 submucosal cancers. We also prepared 200 test images each of 100 cancers from another institution. We designed an AI classifier to differentiate between intramucosal and submucosal cancers by deep learning. We examined the performance of the AI classifier and the majority vote of the endoscopists as high confidence and low confidence diagnostic probability, respectively, and cooperatively combined them to establish a diagnostic method providing high accuracy.

**Results:**

Internal evaluation of the training images showed that accuracy, sensitivity, specificity, and F1 measure by the AI classifier were 77%, 76%, 78%, and 0.768, and those of the majority vote of the endoscopists were 72.6%, 53.6%, 91.6%, and 0.662, respectively. A diagnostic method based on cooperation between AI and the endoscopists showed that the respective values were 78.0%, 76.0%, 80.0%, and 0.776 for the test images. The value of F1 measure was especially higher than those by AI or the endoscopists alone.

**Conclusions:**

Cooperation between AI and endoscopists improved the diagnostic ability to determine invasion depth of early gastric cancer.

**Supplementary Information:**

The online version contains supplementary material available at 10.1007/s10120-022-01330-9.

## Introduction

Gastric cancer is the fourth most common cause of cancer-related deaths in the world [1], but when diagnosed in its early stages, gastric cancer can be cured by endoscopic or surgical resection. According to the Japanese gastric cancer treatment guidelines, (1) differentiated intramucosal cancer without ulceration, (2) differentiated intramucosal cancer within 3 cm in size with ulceration, and (3) undifferentiated intramucosal cancer within 2 cm in size are lesions with an indication for endoscopic resection [2]. For early gastric cancer not meeting these criteria, surgical resection of two-thirds of the stomach is recommended as a standard procedure. Because the extent of resection varies significantly between endoscopic resection and surgical resection, there are large differences in the invasiveness of treatment and post-treatment quality of life. The preoperative diagnosis by endoscopists greatly impacts the treatment outcome of the patients.

The most difficult criterion for the selection of endoscopic resection or surgical resection is differentiation of whether the invasion depth of gastric cancer is intramucosal or submucosal. However, the diagnostic ability to determine whether the cancer is limited within the mucosal layer or is invasive to the submucosal layer is about 70% by endoscopic or endosonographic examination [3–5]. No modality has yet been developed to accurately diagnose the depth of early gastric cancer.

Recently, an artificial intelligence (AI) classifier has been applied to diagnose the invasion depth of gastric cancer [6–8]. Zhu et al. reported that AI-assisted diagnosis of the invasion depth of gastric cancer had a sensitivity of 76.4%, specificity of 95.5%, and accuracy of 89.1% [6]. Nagao et al. showed that their AI classifier using white-light imaging had a sensitivity of 84.4%, specificity of 99.4%, and accuracy of 94.5% [7]. Their diagnostic ability is excellent, but their subjects include those with advanced cancer. We focused on the clinically more difficult differentiation between intramucosal and submucosal gastric cancer.

We designed an AI classifier to differentiate between intramucosal and submucosal gastric cancers. Then, taking into consideration the diagnostic performance of both the AI classifier and endoscopists, we proposed a diagnostic method to determine invasion depth of early gastric cancer based on cooperation between the AI classifier and the majority vote of expert endoscopists.

## Methods

### Definitions of gastric cancer terminology

Macroscopic type, histological type, and invasion depth of gastric cancer were classified based on the classification of the Japanese Gastric Cancer Association [9]. Mainly, depressed-type early gastric cancer lesions were included in this study. Therefore, the macroscopic type was either type 0-IIc or type 0-III. For histological type, well-differentiated adenocarcinoma, moderately differentiated adenocarcinoma, and papillary adenocarcinoma were considered differentiated-type cancer. Poorly differentiated adenocarcinoma, signet-ring cell carcinoma, and mucinous carcinoma were considered undifferentiated-type cancer. When both were mixed, the lesions were classified into the histological type of the predominant lesion. Depth was classified as intramucosal cancer (M), cancer with submucosal (SM) invasion of < 500 μm (SM1), and cancer with submucosal invasion of ≥ 500 μm (SM2).

### Training and test images

A flowchart outlining the study is shown in Supplementary Fig. 1. The Ethics Committees of Yamaguchi University Hospital and Hofu Institute of Gastroenterology approved this study. We retrospectively reviewed the endoscopic images and histopathological diagnoses at Yamaguchi University Hospital from 2009 to October 2020. We selected cases in which the patient underwent endoscopic resection or surgery for depressed-type early gastric cancer. There were 250 training cases each for the intramucosal and submucosal cancers, excluding cases with poor observational conditions, multiple lesions in one image, and lesions that did not fit within one image. We selected one representative white-light image from each case and collected 500 images for training. The endoscopic images and histopathological diagnoses were collected at the Hofu Institute of Gastroenterology from 2007 to January 2017 and reviewed retrospectively. The cases were identified in the same way as those for training. In total, 200 test images were created with 100 cases of intramucosal cancers and 100 cases of submucosal cancers. Both the test and training cases are consecutive cases, and they were not arbitrarily selected. Training and test images were captured using a GIF-H260, GIF-Q260J, GIF-H260Z, GIF-H290, or GIF-H290Z endoscope (Olympus, Tokyo, Japan).

The clinicopathological characteristics of the training cases are shown in Supplementary Table 1. Of the 250 SM cancers, 95 were SM1 cancer and 155 were SM2 cancer. The clinicopathological characteristics of the test cases are shown in Supplementary Table 2. Of the 100 SM cancers, 26 were SM1 cancer, and 74 were SM2 cancer.

### Design of an AI classifier to diagnose invasion depth of early gastric cancer

We used the EfficientnetB1 model for learning, which was pre-trained on ImageNet, a large dataset of more than 14 million images, to design the AI classifier for diagnosing invasion depth of early gastric cancer. The EfficientnetB1 model was used for the feature extraction layer from the input images. The extracted features were replaced by a fully coupled layer to produce two outputs: intramucosal (M) and submucosal (SM) cancers.

Two experienced endoscopists (A.G. and J.N.), certificated member of the Japan Gastroenterological Endoscopy Society, examined all images along with the corresponding macroscopic and histopathology findings and then circled a cancerous area on the individual images. The images were cropped in an outer frame bordering the cancerous area and resized to 240 × 240 pixels. The hardware used to construct the AI classifier included an NVIDIA GeForce RTX 3070 graphics processing unit (GPU) and an AMD Ryzen Threadripper 3960X 24-core central processing unit. The classifier was prepared in the Python 3.8.5 and Tensorflow 2.4.1 environments. The hyperparameters of the classifier were evaluated with a batch size of 32, and the number of epochs was evaluated between 15 and 25, and the 15 with the highest evaluation results were adopted. The fully coupled layer was optimized with the Adam function and a learning rate of 0.001.

### Internal evaluation of diagnostic ability of the AI classifier for training images

The diagnostic ability of the AI classifier to differentiate intramucosal and submucosal cancers was evaluated by the leave-one-out method [10] with 500 training images. According to this method, the 500 training images were divided into 499 training images and one pseudo-test image. We used the 499 training images to design the AI classifier based on deep learning and diagnosed one pseudo-test image. Thus, the danger of an optimistic bias due to the over-fitting the training images may decrease. This step was repeated 500 times such that each of the 500 training images was selected once as a pseudo-test image, and the diagnostic ability of the AI classifier was evaluated internally. Submucosal cancer was defined as positive, and the accuracy, sensitivity, specificity, positive predictive value, negative predictive value, and F1 measure were calculated. The F1 measure is the harmonic mean of sensitivity and PPV, expressed as 2 × sensitivity × PPV/(sensitivity + PPV). We used the F1 measure as a benchmark for making a balanced diagnosis of M and SM cancers.

The softmax function was used to output a continuous value from 0 to 1 for the diagnostic probability of classification as intramucosal (M) or submucosal (SM) cancer. Diagnostic probability of the AI classifier exceeding 75% was defined as high confidence and 51–75% as low confidence, and diagnostic ability between high confidence and low confidence was evaluated.

### Diagnoses by individual endoscopists

Eight endoscopists unaware of the pathology results of the 500 training images were asked to differentiate whether the invasion depth of gastric cancer was intramucosal or submucosal. We mainly use white-light imaging to diagnose the invasion depth of gastric cancer. For mainly depressed lesions, M cancer is characterized by a flat depressed base, and the tip of the converging fold narrows irregularly or is abruptly interrupted. In contrast, SM cancer has an irregular depressed base, and the tip of the converging fold is enlarged [11]. Nagahama et al. also reported that lesions positive for the non-extension sign were classified as SM2 cancers, whereas those negative for the non-extension sign were classified as M-SM1 cancers [12]. The endoscopists diagnose invasion depth of gastric cancer based on these previously published findings. The diagnosis was made by 4 (A–D) experts in esophagogastroduodenoscopy with over 10 years (10–15 years) of experience, and 4 (E–H) endoscopists with less than 5 years (3–5 years) of experience. Submucosal cancer was defined as positive, and the accuracy, sensitivity, specificity, positive predictive value, negative predictive value, and F1 measure was calculated for each endoscopist.

For the decision based on the diagnoses from individual endoscopists, we adopted a majority vote for simplicity. It is well-known in pattern recognition fields that markers which are called features or specific attributes of the patients in diagnosis cannot be selected on the basis of their individual effectiveness [13]. In this study, both AI classifier and endoscopists are considered markers. Thus, all combinations of markers should be paid attention [14]. Considering endoscopists as markers, combinations of 3 out of 4 expert endoscopists were studied. The combination with the highest F1 measure was determined by a majority vote of 3 expert endoscopists. Unanimous voting by all 3 endoscopists was defined as high confidence and other results as low confidence. We evaluated the diagnostic ability between high confidence and low confidence.

### Cooperation between the AI classifier and endoscopists

**Fig. 1:**
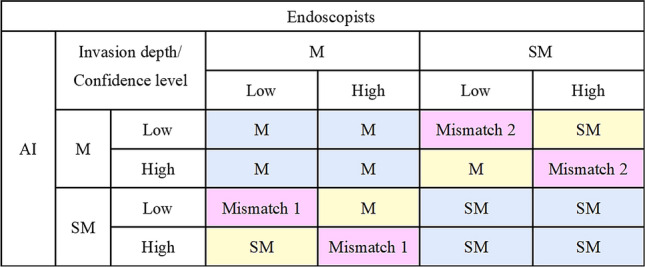
Diagnostic method of determining invasion depth by cooperation between AI and the endoscopists. If the diagnosis by AI and the endoscopists was consistent, the diagnosis was considered final (indicated by the blue cells). If the diagnosis by AI and the endoscopists differed, the diagnosis with the higher confidence level was adopted (indicated by the yellow cells). Diagnoses by AI and the endoscopists that did not agree at the same confidence level are considered mismatches (indicated by the pink cells). M, intramucosal cancer; SM, submucosal invasion; AI, artificial intelligence; Mismatch 1, AI diagnosis is SM but that of the endoscopists is M; Mismatch 2, AI diagnosis is M but that of the endoscopists is SM

To explore how endoscopists can utilize the diagnostic support of the AI classifier in the clinical setting, we devised a diagnostic method based on cooperation between the AI classifier and the endoscopists as shown in Fig. 1. If the diagnoses of AI and the endoscopists were consistent, the diagnosis was considered final (indicated by the blue cells in Fig. 1), If the diagnoses of AI and the endoscopists differed, the diagnosis with the higher confidence level was adopted (indicated by the yellow cells in Fig. 1). If the diagnoses of AI and the endoscopists did not agree at the same confidence level (indicated by the pink cells in Fig. 1), the following four patterns were examined. Pattern I: the AI diagnosis was adopted for both mismatch 1 and 2. Pattern II: the endoscopists’ diagnosis was adopted for mismatch 1, where the diagnosis by AI is SM and by the endoscopists is M, and the AI diagnosis was adopted for mismatch 2, where the diagnosis by AI is M and by the endoscopists is SM. Pattern III: the AI diagnosis was adopted for mismatch 1, where the diagnosis by AI is SM and by the endoscopists is M, and the endoscopists’ diagnosis was adopted for mismatch 2, where the diagnosis by AI is M and by the endoscopists is SM. Pattern IV: the endoscopists’ diagnosis was adopted for both mismatch 1 and 2. The pattern with the best F1 measure on the training images was used as the final diagnosis for cooperation between the AI classifier and the endoscopists in this study.

### Diagnosis of test images

We collected 200 test images of cases from another institution independent of the training cases. The selected endoscopists were also asked to differentiate whether the invasion depth of gastric cancer was intramucosal or submucosal for the test images and then the majority vote was conducted. The accuracy, sensitivity, specificity, positive predictive value, negative predictive value, and F1 measure were calculated for the AI classifier, the endoscopists, and cooperation between the AI classifier and the endoscopists.

### Characteristics of misdiagnosis

Even though the AI classifier performed the diagnosis with high confidence of 95% or more, we presented the misdiagnosed cases and summarized their characteristics.

### Statistical analysis

The data were statistically analyzed using StatFlex V6 statistical software (Artech Co., Ltd., Osaka, Japan). Quantitative variables are presented as median and range, and qualitative variables are presented as frequency and percentage. Comparison of the accuracy between high confidence and low confidence was examined by the chi-square test for 2 × 2 contingency tables.

## Results

### Diagnostic ability by AI and endoscopists for training images

Internal evaluation of training images showed that accuracy, sensitivity, specificity, and F1 measure were 77%, 76%, 78%, and 0.768 by the AI classifier alone. The diagnostic ability by individual endoscopists is shown in Table 1. The diagnostic ability of the expert endoscopists was generally better than that of the other endoscopists. The majority vote of 3 expert endoscopists A, B, and C resulted in the highest F1 measure than for any other combination (Table 2). We, therefore, decided to adopt the majority diagnosis by endoscopists A, B, and C in the following analysis.

**Table 1:** Diagnostic ability by the endoscopists for the training images

	Expert endoscopists					Endoscopists				
	Mean	A	B	C	D	Mean	E	F	G	H
Accuracy, %	68.0	68.8	71.8	66.6	64.8	62.6	62.0	65.2	60.4	62.8
Sensitivity, %	54.8	61.6	54.4	43.2	60.0	54.0	46.8	54.8	52.0	62.4
Specificity, %	81.2	76.0	89.2	90.0	69.6	71.2	77.2	75.6	68.8	63.2
PPV, %	74.5	72.0	83.4	81.2	66.4	65.2	67.2	69.2	62.5	62.9
NPV, %	64.2	66.4	66.2	61.3	63.5	60.8	59.2	62.6	58.9	62.7
F1-measure	0.654	0.664	0.658	0.564	0.63	0.614	0.552	0.612	0.568	0.626

*PPV* positive predictive value, *NPV* negative predictive value

**Table 2:** Diagnostic ability by a majority vote of 3 expert endoscopists for the training images

	Endoscopists			
	A・B・C	A・B・D	A・C・D	B・C・D
Accuracy, %	72.6	67.8	66.8	72.0
Sensitivity, %	53.6	57.2	54.4	54.0
Specificity, %	91.6	78.4	79.2	90.0
PPV, %	86.5	72.6	72.3	84.4
NPV, %	66.4	64.7	63.5	66.2
F1-measure	0.662	0.640	0.621	0.659

*PPV* positive predictive value, *NPV* negative predictive value

The accuracy of the AI classifier with high confidence was 91.0%. The accuracy for low confidence was 73.0%. When the majority vote of endoscopists A, B, and C was unanimous (high confidence), the accuracy was 80.6%, and that with low confidence was 62.2%. Both the AI classifier and the endoscopists had significantly higher accuracy in high confidence cases at *P* < 0.001.

### Cooperation between AI and endoscopists for training images

We proposed a diagnostic method based on cooperation between AI and the endoscopists as shown in Fig. 1. Table 3 shows the results of examining the four possible combinations of the AI and endoscopists’ diagnoses when AI and the endoscopists did not agree at the same confidence level. If we adopted the diagnosis of the AI classifier for mismatch 1 and adopted the endoscopists’ diagnosis for mismatch 2, i.e., Pattern III, the F1 measure was the highest. Thus, we used Pattern III below. Cooperation between the AI classifier and the endoscopists resulted in accuracy, sensitivity, specificity, and F1 measure of 79.6%, 74.4%, 84.8%, and 0.785, respectively.

**Table 3:** Diagnostic ability of possible combinations between the AI classifier and the endoscopists for the training images

	Pattern I	Pattern II	Pattern III	Pattern IV
Accuracy, %	79	75.2	79.6	75.8
Sensitivity, %	69.2	55.6	74.4	60.8
Specificity, %	88.8	94.8	84.8	90.8
PPV, %	86.1	91.4	83	86.9
NPV, %	74.2	68.1	76.8	69.8
F1-measure	0.767	0.692	0.785	0.715

*PPV* positive predictive value, *NPV* negative predictive value

### Diagnostic ability by cooperation between AI classifier and endoscopists for test images

Table 4 shows the diagnostic ability of AI, the endoscopists, and the proposed method based on cooperation between AI and the endoscopists for the test images, respectively. The accuracy, sensitivity, specificity, positive predictive value, negative predictive value, and F1 measure for the AI classifier alone were 72.5%, 74.0%, 71%, 71.8%, 73.2%, and 0.729; those for the endoscopists were 70.0%, 52.0%, 88.0%, 81.3%, 64.7%, and 0.634; and those for cooperation between the AI classifier and the endoscopists were 78.0%, 76.0%, 80.0%, 79.2%, 70.2%, and 0.776, respectively. Cooperation between the AI classifier and the endoscopists improved the diagnostic ability as compared to that by AI or the endoscopists alone.

**Table 4:** Diagnostic ability of the AI classifier, endoscopists, and cooperation between AI and endoscopists for the test images

	AI	Endoscopists	Cooperation between AI and endoscopists
Accuracy, %	72.5	70.0	78.0
Sensitivity, %	74.0	52.0	76.0
Specificity, %	71.0	88.0	80.0
PPV, %	71.8	81.3	79.2
NPV, %	73.2	64.7	70.2
F1-measure	0.729	0.634	0.776

*PPV* positive predictive value, *NPV* negative predictive value

### Characteristics of misdiagnosis

**Fig. 2:**
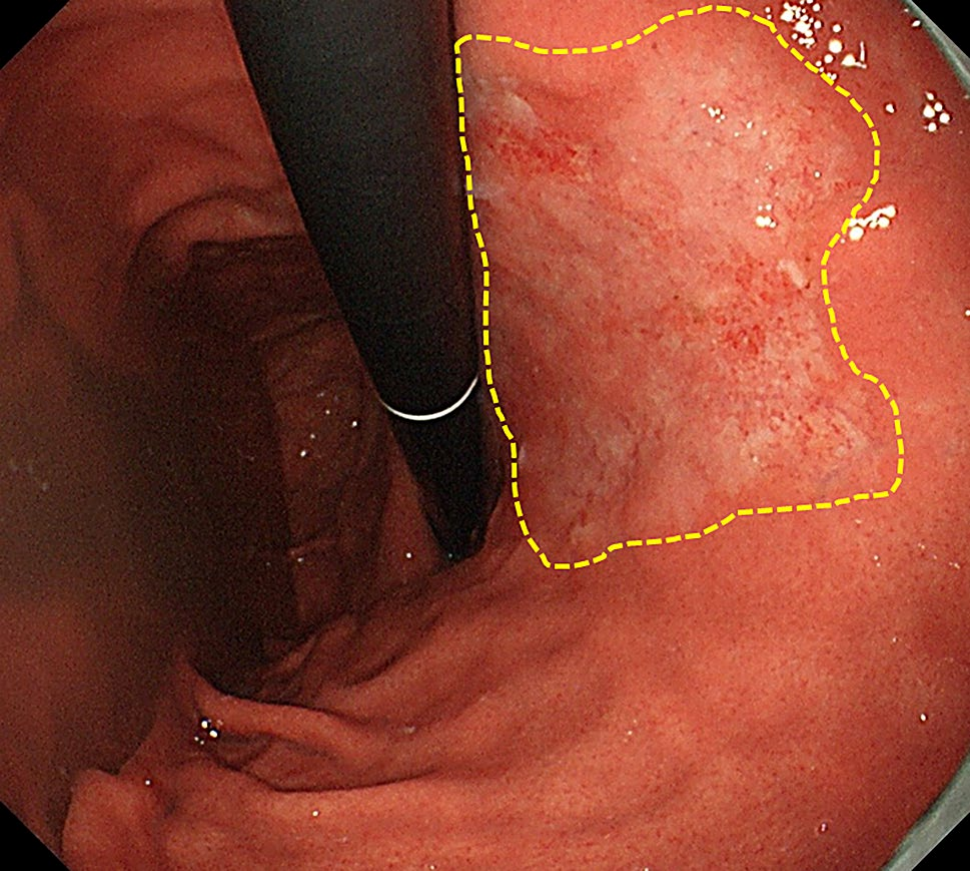
Cases misdiagnosed by AI. Lesser curvature of the gastric body, ⌀44 mm, well-differentiated adenocarcinoma

Five cases of M cancer were misdiagnosed as SM cancer with a diagnostic probability of 95% or more by the AI classifier. Table 3 shows these cases. The tumor diameter was more than 30 mm in all cases. Three of the 5 cases were undifferentiated-type cancer. Case 1 was also misdiagnosed with high confidence by the endoscopists. It was superficial depressed lesion that had spread to the lesser curvature of the stomach body (Fig. 2).

## Discussion

We constructed an AI classifier to differentiate intramucosal and submucosal cancers and explored how the AI classifier can be used to support diagnosis in real-world clinical practice. Waki et al. reported that AI assisted the endoscopist in detecting esophageal cancer by increasing sensitivity without reducing specificity [15]. We combined AI with the diagnosis decided by majority vote of three expert endoscopists to determine the best diagnostic method. We believe that AI should assist endoscopists in improving their diagnostic ability and that accurate diagnosis will lead to better outcomes for the patients. Our proposal to effectively utilize AI together with physicians’ determinations will be used not only for endoscopic diagnosis but also in various other fields.

The AI classifier tended to misdiagnose intramucosal cancer as submucosal cancer, that is, it tended to infer a deeper depth of cancer. In contrast, the endoscopists tended to infer a shallower depth. This may come from a bias inspired by endoscopists’ thoughts to provide minimally invasive care to patients. Endoscopic resection also has clinical roles in providing an accurate diagnosis and reducing unnecessary surgery, whereas the AI classifier has no such bias. Taking advantage of the characteristics of both, we devised a diagnostic method based on cooperation between the AI classifier and endoscopists. Through cooperation with the AI classifier, endoscopists were able to reduce the number of shallow cancer misdiagnoses and improve diagnostic ability. The F1 measure in cooperation between the AI classifier and endoscopists was especially higher than those by AI or the endoscopists alone. The F1 measure, which was used as the indicator of diagnosability, is considered an accurate indicator focusing not only on the sensitivity but also on the positive predictive value. Endoscopists should discuss the diagnosis of each case at a conference and take into account the AI classifier’s decision so as to diagnose the invasion depth as accurately as possible.

The reports of Zhu et al. [6] and Nagao et al. [7] were based on cases that included some advanced cancer and, therefore, the diagnostic abilities of their AI were probably better than that of our AI classifier. We focused only on patients with early gastric cancer and aimed to differentiate M cancer, an absolute indication for endoscopic submucosal dissection (ESD), from SM cancer, which is not an absolute indication for ESD, following the Japanese Gastric Cancer Treatment Guidelines. This study is also the first trial to evaluate the ability of the AI classifier in diagnosis of the invasion depth with cases from another institution that were independent of the training cases. The diagnostic ability of our AI classifier alone for the test images was almost comparable to that by internal evaluation from training cases. This suggested that training of our AI classifier was adequate.

We studied the misdiagnosed cases even though the diagnostic probability was more than 95% by the AI classifier. In all of these cases, intramucosal cancer was misdiagnosed as submucosal cancer. All tumors were more than 30 mm in diameter, and 3 of the 5 cases were of undifferentiated-type cancer. Thus, the diagnostic accuracy of the AI classifier would appear to be affected by size and differentiation of the tumor. We used the images cropped in an outer frame bordering the cancerous area and resized the image to 240 × 240 pixels, and thus the AI classifier might not be able to consider tumor size as a feature in diagnosis of the invasion depth. Yoon et al. reported that diagnosis of the invasion depth of undifferentiated-type gastric cancer was more difficult than that of differentiated-type gastric cancer [16]. As undifferentiated-type intramucosal cancer within 2 cm become a new indication for ESD [2, 17], the invasion depth of undifferentiated-type cancer must be accurately diagnosed prior to treatment. The endoscopists tended to infer a shallower depth, resulting in a high specificity (sensitivity to diagnose M cancer), but the specificity has been reduced when combined with results of AI. The risk of overtreatment due to decreased specificity must be taken into account. We believe that AI specificity can be improved by learning from additional gastric cancer cases of undifferentiated-type and large tumor diameters that are contributing to misdiagnosis.

A limitation of this study is that it included only depressed-type early gastric cancer, and protruded-type lesions were not examined. Also, only two institutions participated in the study. A multicenter study is needed to further validate the usefulness of our method.

## Conclusion

We devised a diagnostic method based on cooperation between our AI classifier and endoscopists. Cooperation between AI and endoscopists improved diagnostic ability for the invasion depth of early gastric cancer.

## Supplementary Information

Below is the link to the electronic supplementary material.

Supplementary file1 (TIF 128 KB)

Supplementary file2 (DOCX 27 KB)
