# Supplementary figures and images for: Cooperation between artificial intelligence and endoscopists for diagnosing invasion depth of early gastric cancer
Source: Gastric Cancer. 2022 Aug 30;26(1):116–22. doi: 10.1007/s10120-022-01330-9 (PMC9813068; doi:10.1007/s10120-022-01330-9)

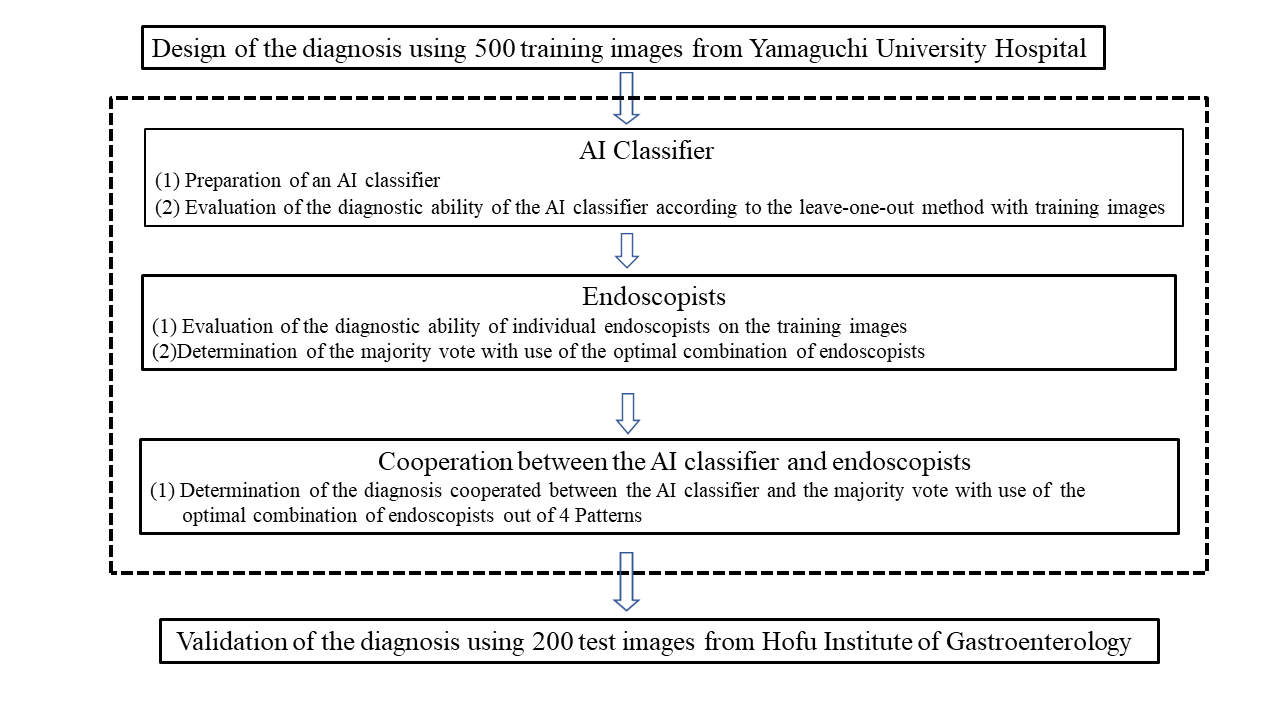

Supplement: Supplementary file 1 — Supplementary file1 (TIF 128 KB) [file 10120_2022_1330_MOESM1_ESM.tif]
