# Supplementary material for: Cooperation between artificial intelligence and endoscopists for diagnosing invasion depth of early gastric cancer
Source: Gastric Cancer. 2022 Aug 30;26(1):116–22. doi: 10.1007/s10120-022-01330-9 (PMC9813068; doi:10.1007/s10120-022-01330-9)
Supplement: Supplementary file 2 — Supplementary file2 (DOCX 27 KB) [file 10120_2022_1330_MOESM2_ESM.docx]

| **Supplementary Table 1** Clinicopathological characteristics of the patients whose images were used for training. | | | | |
| --- | --- | --- | --- | --- |
| **Characteristic** | **M (n=250)** | **SM (n=250)**  **(SM1 95, SM2 155)** | **95%CI** | **p-Value** |
| Age, mean (range), years | 70.5 (47–91) | 70.9 (34–90) | -2.567 - 0.783 | 0.296 |
| Sex |  |  |  |  |
| Male, n (%) | 190 (76) | 191 (76) |  | 0.916 |
| Female, n (%) | 60 (24) | 59 (24) |  |  |
| Treatment |  |  |  |  |
| Endoscopic resection, n (%) | 238 (95) | 115 (46) |  | <0.001 |
| Surgical resection, n (%) | 12 (5) | 135 (54) |  |  |
| Tumor diameter, mean (range), mm | 15.0 (3–55) | 23.1 (5–65) | -10.03 - -6.253 | <0.001 |
| Macroscopic type |  |  |  |  |
| 0-IIc, n (%) | 248 (99) | 196 (78) |  | <0.001 |
| 0-III, n (%) | 2 (1) | 54 (22) |  |  |
| Differentiated type |  |  |  |  |
| Differentiated, n (%) | 242 (97) | 189 (76) |  | <0.001 |
| Undifferentiated, n (%) | 8 (3) | 61 (24) |  |  |
| M, intramucosal cancer; SM, submucosal invasion. | | | | |

| **Supplementary Table 2** Clinicopathological characteristics of the patients whose images were used for testing. | | | | |
| --- | --- | --- | --- | --- |
| **Characteristic** | **M (n=100)** | **SM (n=100)**  **(SM1 26, SM2 74)** | **95%CI** | **p-Value** |
| Age, mean (range), years | 66.5 (28–85) | 70.9 (35–95) | -7.368 - -1.412 | 0.004 |
| Sex |  |  |  |  |
| Male, n (%) | 70 (70) | 74 (74) |  | 0.529 |
| Female, n (%) | 30 (30) | 26 (26) |  |  |
| Treatment |  |  |  |  |
| Endoscopic resection, n (%) | 88 (88) | 45 (45) |  | <0.001 |
| Surgical resection, n (%) | 12 (12) | 55 (55) |  |  |
| Tumor diameter, mean (range), mm | 18.2 (5–80) | 29.3 (4–105) | -15.93 - -6.313 | <0.001 |
| Macroscopic type |  |  |  |  |
| 0-IIc, n (%) | 96 (96) | 79 (79) |  | <0.001 |
| 0-III, n (%) | 4 (4) | 21 (21) |  |  |
| Differentiated type |  |  |  |  |
| Differentiated, n (%) | 72 (72) | 69 (69) |  | 0.642 |
| Undifferentiated, n (%) | 28 (28) | 31 (31) |  |  |
| M, intramucosal cancer; SM, submucosal invasion. | | | | |

| **Supplementary Table 3** Cases misdiagnosed by the AI classifier with a diagnostic probability of 95% or more. | | | | | | | | |
| --- | --- | --- | --- | --- | --- | --- | --- | --- |
| **No.** | **Depth** | **Age** | **Sex** | **Treatment** | **Tumor diameter** | **Macroscopic type** | **Differentiated type** | **Diagnostic probability** |
| 1 | M | 63 | Male | ESD | 44 | 0-IIc | tub1 | 97.2 |
| 2 | M | 76 | Male | ESD | 34 | 0-III | tub2 | 98.7 |
| 3 | M | 28 | Male | ESD | 35 | 0-IIc | sig | 96.0 |
| 4 | M | 58 | Male | Surgical | 80 | 0-IIc | por | 97.5 |
| 5 | M | 73 | Male | Surgical | 41 | 0-IIc | por | 99.5 |
| M, intramucosal; ESD, endoscopic submucosal dissection. | | | | | | | | |
